# Methodological support for the further abstraction of and philosophical examination of empirical findings in the context of caring science

**DOI:** 10.3402/qhw.v11.30482

**Published:** 2016-02-26

**Authors:** Elisabeth Lindberg, Sofia A. Österberg, Ulrica Hörberg

**Affiliations:** 1 Faculty of Caring Science, Work Life and Social Welfare, University of Borås, Borås, Sweden; 2 Department of Health and Caring Sciences, Faculty of Health and Life Sciences, Linnaeus University, Växjö, Sweden

**Keywords:** Philosophical examination, methodological support, reflective lifeworld research, phenomenology, caring science

## Abstract

Phenomena in caring science are often complex and laden with meanings. Empirical research with the aim of capturing lived experiences is one way of revealing the complexity. Sometimes, however, results from empirical research need to be further discussed. One way is to further abstract the result and/or philosophically examine it. This has previously been performed and presented in scientific journals and doctoral theses, contributing to a greater understanding of phenomena in caring science. Although the intentions in many of these publications are laudable, the lack of methodological descriptions as well as a theoretical and systematic foundation can contribute to an ambiguity concerning how the results have emerged during the analysis. The aim of this paper is to describe the methodological support for the further abstraction of and/or philosophical examination of empirical findings. When trying to systematize the support procedures, we have used a reflective lifeworld research (RLR) approach. Based on the assumptions in RLR, this article will present methodological support for a theoretical examination that can include two stages. In the first stage, data from several (two or more) empirical results on an essential level are synthesized into a general structure. Sometimes the analysis ends with the general structure, but sometimes there is a need to proceed further. The second stage can then be a philosophical examination, in which the general structure is discussed in relation to a philosophical text, theory, or concept. It is important that the theories are brought in as the final stage after the completion of the analysis. Core dimensions of the described methodological support are, in accordance with RLR, openness, bridling, and reflection. The methodological support cannot be understood as fixed stages, but rather as a guiding light in the search for further meanings.

The aim of this article is to describe the methodological support for the further abstraction of and philosophical examination of empirical findings. In this article, concepts such as theoretical examination, general structure, and philosophical examination are used. In a theoretical examination, a theory or philosophy contributes to gain greater understanding of the result. In this article, the concept includes a broad perspective on which empirical research is related to philosophy and theory. The methodological support proposed here is a general structure and/or a philosophical examination, which exemplifies variations of how empirical material can be abstracted using reflective lifeworld research (RLR).

Human existence involves a complex variety of intertwined dimensions. Life is lived in an environment characterized by contention, in which biological as well as lived experiences contribute to developing the existential conditions. Caring science has developed a perspective of human existence, especially in relation to health and illness, where the patient's lifeworld is the core dimension. In the present article, a phenomenological lifeworld perspective creates the foundation for caring science as well as for the research approach. Caring science, based on a lifeworld perspective, takes into account the patients’ perspective; how, for example, health, illness, and well-being are experienced by humans. Caring science, based on a lifeworld perspective, also considers humans as a multifaceted whole, where dimensions such as body, soul, space, and time are intertwined and inseparable (Dahlberg, Todres, & Galvin, 2009; Galvin & Todres, 2009, 2013; Todres, Galvin, & Dahlberg, 2014). In the tradition of phenomenology, existential philosophy focuses on what it is like to be human, and how meaning can be found in the ambiguity of life and contribute to draw attention to the vulnerability of human existence (Dahlberg et al., 2009; Galvin & Todres, 2013; Todres et al., 2014). This underpinning links existential philosophy to caring science and contributes to greater insights in the patient perspective.

Describing human existence is a complex issue, and sometimes the researcher concludes with questions that cannot easily be answered by further empirical research. In these cases, philosophy can be one way to gain a greater understanding. In particular, “the philosophy of existence,” which focuses on the meaning of life and existence, can be seen as important for gaining a greater understanding of research findings within the field of caring science. This is due to the possibility of it enriching the phenomena in this field. On the other hand, a risk when performing a philosophical examination without scientific rigor is that the new result can become mundane or plain simple. It can lead to misinterpretations and leave the reader clueless as to how to use the results. In the worst case scenario, it can lower the overall quality.

Reviewing literature with the intention of intertwining results from empirical research together with philosophy reveals a variety in terms of the aim of these theoretical examinations, as well as the methodological performance. The aim of the theoretical paper is described as a way of seeing the results from empirical research in a new light in the literature (Beedholm, Lomborg, & Frederiksen, 2014; Hörberg & Dahlberg, 2015; Lindberg, Ekebergh, Persson, & Hörberg, 2015; Karlsson, Ekebergh, Larsson Mauléon, & Almerud Österberg, 2012) or contributing to further examination of the phenomenon (Evans, Glass, & Traynor, 2014; Öresland, Lutzén, Norberg, Rasmussen, & Määttä, 2013; Rydeman, Törnkvist, Agreus, & Dahlberg, 2012). Some researchers select concepts from philosophy and link these to other concepts not primarily from either philosophy or caring (c.f. Öresland et al., 2013). Others add a common philosophical foundation to manage different ontologies for different professions (c.f. Kane, 2014), discuss basic assumptions for their own research (Routledge, 2007), or create new models based on philosophical theories (c.f. Anderson & Whall, 2013). The philosophy can help describe the full richness and contribute to broadening the horizons of the phenomenon in focus, regardless of the intention. In addition to being presented in scientific journals, the theoretical examination can be part of a doctoral thesis. It can be in the form of a chapter (Berglund, 2011), of a study in a dissertation (Ozolins, 2011), or of an excursus in a monograph (Hörberg, 2008). A theoretical examination can also be a part of a thesis (Palmér, 2015) or included as an article in a compilation thesis (Almerud, 2007; Karlsson, 2013; Lindberg, 2014). Furthermore, it can be a part of the discussion in a monograph (Summer Meranius, 2010; Syrén, 2010).

In the field of caring science, theoretical examinations in the form of a general structure performed together with a philosophical examination (Dahlberg, Dahlberg, & Nyström, 2008) have become a way of gaining a greater understanding of phenomena in caring. However, when reviewing published research claiming to be further abstractions of empirical results, for example, a general structure and/or a philosophical examination, a lack of clarity regarding the methodology can be found. This can be seen in terms of how the selection of philosophical texts has been carried out and how the research results and philosophy have been intertwined in the analysis. All the objectives for conducting theoretical examinations presented above are commendable, but sometimes the lack of methodological descriptions contributes to a potential risk of the validity of the research. There is also often a lack of critical, problem-oriented reflection on the value of the philosophical examination as well as on the performance of the analysis.

The lack of a theoretical and systematic foundation when performing a philosophical examination threatens to undermine the very foundations of the scientific value. Lack of support in methodological and scientific references contributes to an unhelpful ambiguity concerning how the result has emerged during the analysis. A lack of methodological references can also contribute to a certain degree of subjectivity, which in turn undermines the scientific value and contributes to a risk of being questioned in scientific contexts. On the other hand, comprehensive descriptions create a risk of diverting the focus from the actual result of the general structure or philosophical examination. Both extremes in terms of excessively vague and far too detailed descriptions of methods also create a risk for the philosophical examination of being brought into a “scientific no-man's land” between “naïve” philosophy and incomprehensible empirical material or data findings. By clarifying the scientific approach and methodology, the philosophical examination can find its own place in caring science in a more evident manner.

In this article, we will present methodological support for a theoretical examination that can include two stages. The first contains a synthesizing of the results from two or more empirical studies, which results in a general structure (this can be sufficient in some cases). The second consists of a philosophical examination of the general structure in order to further examine the understanding of the phenomenon. The method is derived from the RLR approach developed by Dahlberg et al. (2008) that has been used as the theoretical framework in both general structures and philosophical examinations. Dahlberg et al. (2008) maintain that philosophy and theories cannot be included in the analysis process in phenomenological studies. However, philosophy and theories can contribute to a greater understanding of the phenomenon in focus if the external material is first imported after the full empirical analysis. We maintain that RLR can serve as a theoretical and methodological foundation for philosophical examinations. Methodological support for strengthening researchers in the analysis process is thus needed.

## Aim

The aim of this paper is to describe the methodological support for the further abstraction of and/or philosophical examination of empirical findings.

## RLR approach

RLR is a research approach developed by Dahlberg (Dahlberg, 2006a, 2006b; Dahlberg et al., 2008; Dahlberg & Dahlberg, 2003, 2004). RLR is based on the continental philosophy foremostly derived from Husserl (1970/1936, 1977/1929) and Merleau-Ponty (1968/1964, 2011/1945) and mainly developed during the first half of the twentieth century.

RLR draws on the epistemological understanding of Husserl's lifeworld theory (1970/1936) and the theory of intentionality (1977/1929). Husserl describes the lifeworld as a lived personal world, not possible to withdraw from. We cannot be separated from our lifeworld because we live in and through it. Basically, we are in a natural attitude to the world, which means that it is taken for granted and we thus lack distance to the lifeworld and our experience. In the theory of intentionality, Husserl describes that the consciousness in its basic mode is always directed outwards, towards something else other than oneself. It is first when we become aware of that and how we experience something we can distance ourselves from what we experience. Merleau-Ponty (2011/1945) has further developed Husserl's lifeworld theory and clarified a human being's existence in the world as a “lived body,” an integrated whole where there is no dividing line between body and soul. Merleau-Ponty (1968/1964) has also described the theory of “flesh” (the flesh of the world) where he shows how both existences and matter are affected by the same world in reversibility and share a common world connected with each other (Dahlberg et al., 2008). Dahlberg (2011, 2013) has, based on a phenomenological epistemology, developed not only RLR, but also caring science based on lifeworld theory. This common ground involves concepts with meaning in research as well as in caring, for example a reflective attitude.

The epistemological understanding, together with the methodological ways of thinking, is important for a researcher when applying RLR. When the latter is used, the goal is to discover, analyze, clarify, understand, and describe meanings of phenomena. This means to describe and explore the essential meanings of the phenomenon in focus, that is, what makes this phenomenon the phenomenon that it is, and not anything else (Dahlberg et al., 2008).

RLR should not be understood as a method with rigid (inflexible) predetermined stages, it instead contains methodological principles such as openness, flexibility, and bridling. These are inspired by Gadamer's (2013/1960) skepticism against rigorous and fixed methods in human science. Adopting an openness and flexibility towards the studied phenomenon in focus is of importance for seeing and understanding the phenomenon in a new way (Dahlberg et al., 2008). It is also of importance to adopt a reflective attitude that entails not understanding the meanings of the phenomena too quickly and in an unreflected way. This reflective attitude has been developed by Dahlberg and is described as a bridling of the whole process of understanding (Dahlberg et al., 2008; Dahlberg & Dahlberg, 2003). Bridling one's understanding means to slow down the natural process of understanding, and to be careful to not to be too quick to make definite what is indefinite in order to find the actual presentations as well as the appresentations (Dahlberg et al., 2008; Dahlberg & Dahlberg, 2003). With support of the aforementioned methodological principles, Dahlberg et al. (2008) maintain that the phenomenological analysis starts with a search for meanings of the phenomenon in the data. Related meanings are then grouped together in clusters. The essential meanings gradually emerge through a search for patterns of meanings within and between the clusters that describe the phenomenon in focus. The basic work of the analysis can be described in terms of “figure and background” (Dahlberg, 2011) and has its foundation in Merleau-Ponty's (1968/1964) theory of “the flesh” and “reversibility.” With the support of Merleau-Ponty (1968/1964), Dahlberg et al. (2008) express “that a phenomenon's totality is of *its* particulars, or reverse: a phenomenon's particulars are of *its* totality” (p. 250).

## Methodological support for the further abstraction of and/or philosophical examination of empirical findings

A well-reflected theoretical ground is essential and in phenomenologically guided research a well-defined, clearly expressed phenomenon is something to aim for. This is important regardless of whether the research is empirical or theoretical. The phenomenon can, for example, be derived from new research questions related to previous empirical research. In this article, we use an example from empirical research focusing on the phenomenon of older patients’ participation in team meetings. These studies contain descriptions from the patients’ perspective (Lindberg, Hörberg, Persson, & Ekebergh, 2013) and that of the nurses (Lindberg, Persson, Hörberg, & Ekebergh, 2013). In order to increase the patients’ involvement in their care, the older patient was invited to participate in a team meeting. The team meeting was a way of developing the ward round (Lindberg, Hörberg, et al., 2013; Lindberg, Persson, et al., 2013; Lindberg et al., 2015). New questions emerged after completion of the studies concerning the older patients’ presence rather than participation during the team meetings. Examples of new research questions were viewed against the background of a new meaning structure: How does the presence of older patients at the team meeting manifest itself when the two results are understood in relation to each other? How can the patient's presence at the team meeting be understood at a deeper level? What does the patient's presence mean for aspects of interpersonal relations during the team meeting? Based on these new questions, a new phenomenon was formulated: The older patients’ presence at a team meeting in a ward for older patients. The nature of these “new” questions contributed to a confirmation that the philosophy provided depth, and furthered a greater understanding of the phenomenon (Lindberg et al., 2015). An overview of questions to consider prior to conducting a theoretical examination is presented in Table I, which also includes a brief summary of the methodological support presented in the forthcoming text.

**Table I:** Frame for support when conducting further abstraction or philosophical examination.

The aim of this frame is to provide a brief overview of some of the considerations that have to be made during the process. As previously explained, there are no fixed stages and the general structure as well as the philosophical examination have to be conducted with an attitude of bridling, openness, and reflection
***Questions to consider before conducting further abstraction or philosophical examination***
How can further abstraction and/or philosophy develop your result? Which parts of your results could be further developed by a philosophical examination? Which philosopher would be useful? What is the phenomenon you want to further understand? How can a philosophical examination be justified in your study or thesis?
***Formulating/identifying a phenomenon***
Which questions arise from the studies you want to highlight? What is the phenomenon you want to further understand?
**Methodological support principles**
***General Structure***
Readings of results from empirical studies with an open attitude The results merge together into a new whole … … which can be done by asking questions, discussing what appears to be obvious, as well as the more latent meanings Using figure and background to find new patterns of meanings
***Philosophical examination***
Selected parts of structures of meanings from the general structure are discussed in the light of a philosophical text (or concept) … … this is a process of deep reflection, in which the understanding of the phenomenon can be developed through philosophy The general structure and the philosophical texts can, alternately, appear as figure and background … … which can be done by asking questions, discussing what appears to be obvious, as well as the more latent meanings A new understanding of the phenomenon can emerge resulting in descriptions of meaning structures

### General structure

The data for a general structure consists of two or more empirical results on an essential level of abstraction. Studies conducted with a phenomenological approach are in focus in this article where the important issue is a search for meaning in the results. The essential meanings (patterns or structures of essential meanings) are in focus for the analysis in phenomenological studies, while descriptions in other qualitative methodologies are on the level of meanings.

The analysis process begins with readings of the results with an open attitude, which are merged together to create a new foundation with new structures of meanings on an abstract level. This process does not entail a reanalysis of the data, but is more of a fusion between, and an abstraction of, previous results. Further development of the understanding of the phenomenon in focus, which is guided by research questions, is possible by relating the two (or more) essences to each other by using “figure and background” where meanings (or patterns of meanings) from one of the essences is placed as a figure that can be seen to stand out against a background, the meanings (or patterns of meanings) from the other essences and vice versa. The intention is to find new intertwined patterns of meanings of the studied phenomenon by attempting to understand the primary results in relation to each other (supported by “figure and background”). Dahlberg et al. (2008) point out the importance of working actively with figure and background and to highlight all the possible meanings in relation to each other in different combinations. Openness, flexibility, and bridling must be present throughout the process. This approach if handled carefully and with respect can contribute to furthering the understanding and to merging the results onto a new and more abstract level. A general structure is formulated that is based on the variations between the parts and the whole, and with support from the phenomenon and research questions. An example of the process is described in Table II.

**Table II:** Examples from the creation of the general structure.

Excerpt from essence study I Patients’ perspective	Excerpt from essence study II Nurses’ perspective	General structure
The team meeting is an emotional meeting concerned with life and existence.	Participation is challenged by the patients’ vulnerability and by the subordinated role assigned to the patient.	Participation and invitation can turn into loneliness and give rise to feelings of abandonment, and feelings of being neglected and invisible.
“Real life” takes a break when hospitalization occurs. Freedom and independence in everyday life is bracketed when one needs to surrender to the care of others and enter into a patient role.	Patient participation affects the relationship between the professionals and the patient perspective is challenged by the professionals’ need for maintaining familiar patterns.	Going beyond familiar borders, as well as working to create conditions for participation for everyone present, involves the risk of being excluded from the companionship.

Source: Lindberg et al. 2015.

The process sometimes ends at this point. The general structure has taken the research far enough. In other cases, the phenomenon asks for more in order to be fully understood. This is where the second stage in the analysis process begins.

### Philosophical examination

In order to gain a greater understanding of the phenomenon, a philosophical examination of the general structure can be carried out. A general structure is abstract and general. Philosophy can therefore be applied and increase the value of the general structure. Dahlberg (2013) describes the philosophy in philosophical examinations of empirical findings as working “like a giant spotlight, illuminating all dark spots of the empirical description” (p. 39). The creation of a philosophical examination can be performed through discussing the general structure in the light of a philosophical text, a theory, or a concept. The encounter between the patterns (or structures) of meanings in the general structure and the philosophical texts generates a powerful process, which needs to be open and reflected upon during the entire process. This analysis can be understood in terms of “figure and background.” Parts of the general structure (or patterns of meanings in the general structure) can be seen to stand out against its background, in this case parts of the philosophical text. Dahlberg (2011) states that the analysis work of “figure and background” entails playing with different meanings that are present, in this case in the general structure and the philosophical text. In the encounter between the meanings of the phenomenon in focus and the meanings of the philosophical texts, new meanings and a deeper understanding of the phenomenon can emerge. It concerns allowing the general structure to be intertwined with a philosophy, a concept, or theory.

Asking questions of the text is a means of retaining transparency and encouraging reflection. Examples of such questions are: How can we understand more of the findings with the support of the philosophy? How can the meaning of illness be understood in relation to the existential meanings of being a human? How can the meanings in the philosophical text enrich the understanding of the phenomenon?

The selection of philosophy, concept, or theory cannot be made randomly or by just “bumping in” to something that with a quick gaze can appear to verify the general structure. The process of fusion is far more complex and needs to be discussed. In this creative act, the researcher needs to pay careful attention throughout the entire process. Otherwise the general structure or the philosophical parts could obscure each other. The reflective attitude, described by Dahlberg and Dahlberg (2003) and Dahlberg et al. (2008) as a bridling of the whole understanding process, is of great importance here.

In this process, a new understanding can emerge resulting in meaning structures, which can be seen as a type of constituent of the general structure intertwined with the philosophy. The goal is to generate new knowledge and to reach a new understanding, rather than to explain. In the absence of methodological literature, the term “meaning structures” is a way of highlighting both the intention of a search for meaning as well as the need for a structure of the presentation. An example of the process from the studies conducted by Lindberg et al. (2015) is described in Table III.

**Table III:** Examples from the creation of the philosophical examination.

General structure	Meaning structure	Viewed against the philosophy
Participation and invitation can turn into loneliness and give rise to feelings of abandonment, and feelings of being neglected and invisible.	Mood as a force in existence	In Heidegger's philosophy, mood is something that is always present; man is “tuned” in its existence. Unlike emotions, which are more related to events and thoughts, the mood is already present. The mood contributes to a closeness of emotions. In dark moments, loneliness, vulnerability and the finitude of life paralyze, and in other moments, joy and gratitude create happiness and a will to live. And in the often formal structure of the team meeting, this proximity to emotions contributes to both a sense of loss over how to handle the emotions, as well as to a feeling of warmth and thoughtfulness in the situation.
Going beyond familiar borders, as well as working to create conditions for participation for everyone present, involves the risk of being excluded from the companionship.	Loneliness in the presence of others	Situations arise, during the team meeting, in which the participants’ vulnerability becomes obvious. Through interest and curiosity for the other, possible tensions can be overcome, but by maintaining locked positions and by a lack of knowledge, the professionals may also give themselves a mandate to an interpretative privilege of the others’ experiences. Heidegger (1962/1927, p. 157) argues that loneliness is a form of, what in Heidegger's philosophy is termed as “Being-with”; “The Other can be missing only in and for a Being-with.” The ambiguity of existence emerges in the variations of loneliness and “Being-with” in which humans find themselves in.

Source: Lindberg et al., 2015.

## Concluding reflections

The goal in RLR is to be as open and flexible as possible towards that which shows itself during the process of analyzing. Thoughtfulness and bridling are core dimensions in theoretical examinations. A bridled understanding can be seen as an attempt, in a thoughtful and reflective way, to try to see more than what is obviously seen (Dahlberg et al., 2008). Dahlberg (2011) states that “bridling is the main phenomenological answer to the questions of validity and objectivity” (p. 28) and further that researchers must have a scientific (bridled) attitude activated throughout the whole research process. It is thus important to describe clearly the research process in philosophical examinations, that is., motivate the choice of philosophical texts; transparency concerning which data (empirical findings) is the basis for the analysis; carefully describe the analysis process and the presentation of results; and describe how the process of understanding has been bridled.It is otherwise difficult to argue for validity and objectivity in a study that utilizes philosophical examinations.

There is a need to have something to aim for; otherwise the research is at risk of becoming a “fuzzy mess.” We have used RLR, described by Dahlberg et al. (2008), when trying to systematize procedures of support. We have also found it fruitful to work with original texts from Husserl (1970/193, 1977/1929) and Merleau-Ponty (2011/1945, 1968/1964) in order to gain a greater understanding. Research findings should be trustworthy and every research study must be evaluated in relation to the procedures used to generate the findings (Polit & Beck, 2006). This applies not only to empirical research, but also when empirical findings are examined philosophically. We argue that methodological considerations (for example, validity and generalization) need to be reflected upon when philosophical examinations are conducted. In our case, the methodological principles derive from Dahlberg et al. (2008), but as RLR is ontologically and epistemologically grounded in the work of Husserl and Merleau-Ponty, we have used selected parts of the philosophers’ work in order to better understand the theoretical ground. Dahlberg et al. (2008) clarifies that a generalization of results requires that these are lifted above a concrete level to a more abstract level. A general structure with its invariant meaning structure and abstract level can be generalized to similar phenomena and context. On the other hand, the philosophical examination can contribute to gain a greater understanding of the phenomenon in focus for the study and thus enable generalization. At the same time, we want to emphasize that the overall purpose with philosophical examinations is to gain a greater understanding of complex phenomena and in accordance with Dahlberg's (2013) term; like a spotlight to illuminate dark spots in empirical findings.

Philosophy can then be a way of gaining a greater understanding of what the phenomenological analysis has already proven, or described in another way; philosophy can open one's eyes for aspects that otherwise had passed by unseen. It is important that the theories are set as the final stage after the analysis is completed. Following this, the analysis of the empirical material must be completed before the result is intertwined with philosophy. As pointed out earlier, a philosophical examination can be conducted from studies inspired by phenomenology (as in our example), but a philosophical examination can also be conducted from other forms of results. Rydeman et al. (2012), for example, used RLR as way to further interpret empirical data with lifeworld theory. Dahlberg et al. (2008) point out that “the included theory should illuminate the findings and not data. If the latter was the case, it would be an interpretative analysis” (p. 273). This approach differs from methodological principles inspired by hermeneutics. It also differs from a secondary analysis in which the empirical material is reanalyzed.

Phenomenological research can sometimes be an act of balancing between science and art. According to this, it is important to describe the results in such a way as to strike a chord with the reader. The reader may feel that the contents both concern and are relevant for humans (Finlay, 2011). This “extra dimension” of scientific approach can be challenging in relation to traditional structures for scientific work. In order for meaning to appear, humans need to be concerned. A scientific text sometimes needs creativity in order to touch the reader, but not in a way that makes it insignificant. This balancing act is important when conducting theoretical examinations based on phenomenology. The scientific tradition, which phenomenology wants to go beyond, has influenced language. Describing new phenomena with old words and expressions is limiting, and methodological creativity can thus be useful. The methodology has, however, to be valid and trustworthy. Even so, it is a possibility. In order to make use of the full potential of the language, metaphors can contribute to describe certain dimensions of the phenomenon under investigation. Figurative descriptions can contribute to make visible what has not previously been seen. However, every metaphor and description has to be chosen very carefully and used “with full respect to the phenomenon” (c.f. Dahlberg & Dahlberg, 2004, p. 272).

In the examples from older patients’ participation in team meetings, philosophy contributed to further expanding insights (Lindberg et al., 2015). This approach contributed to greater knowledge in a different way than would have been possible in another empirical study. The research questions that emerged from the results of the empirical studies gave implications of existential dimensions, which would have been hard to grasp and articulate without philosophy. The referred study contained both a general structure and a philosophical examination, which was due to the phenomenon and research questions. However, as previously described it is not necessary to conduct both steps, as sometimes a general structure or a philosophical examination can suffice.
